# Role of Radiation Therapy in the Management of Renal Cell Cancer

**DOI:** 10.3390/cancers3044010

**Published:** 2011-10-26

**Authors:** Angel I. Blanco, Bin S. Teh, Robert J. Amato

**Affiliations:** 1 Department of Radiation Oncology, The Methodist Hospital, The Methodist Hospital Research Institute, Houston, TX 77030, USA; E-Mails: aiblanco@tmhs.org (A.I.B.); bteh@tmhs.org (B.S.T.); 2 Department of Radiation Oncology, The Methodist Hospital, Houston, TX 77030, USA; 3 Division of Oncology, University of Texas Health Science Center at Houston, Memorial Hermann Cancer Center, Houston, TX 77030, USA

**Keywords:** radiation therapy, stereotactic radiosurgery, renal cell carcinoma

## Abstract

Renal cell carcinoma (RCC) is traditionally considered to be radioresistant; therefore, conventional radiotherapy (RT) fraction sizes of 1.8 to 2 Gy are thought to have little role in the management of primary RCC, especially for curative disease. In the setting of metastatic RCC, conventionally fractionated RT has been an effective palliative treatment in 50% of patients. Recent technological advances in radiation oncology have led to the clinical implementation of image-guided radiotherapy, allowing biologically potent doses to the tumors intra- and extra-cranially. As predicted by radiobiologic modeling, favorable outcomes have been observed with highly hypofractionated schemes modeled after the experience with intracranial stereotactic radiosurgery (SRS) for RCC brain metastases with reported local control rates averaging 85%. At present, both primary and metastatic RCC tumors may be successfully treated using stereotactic approaches, which utilize steep dose gradients to maximally preserve function and avoid toxicity of adjacent organs including liver, uninvolved kidney, bowel, and spinal cord regions. Future endeavors will combine stereotactic body radiation therapy (SBRT) with novel targeted therapies, such as tyrosine kinase inhibitors and targeted rapamycin (mTOR) inhibitors, to maximize both local and systemic control.

## 1. Introduction

An estimated 60,920 new cases of kidney cancer are expected to be diagnosed in the United States in 2011, of which the majority (>90%) will be renal cell carcinoma (RCC). RCC incidence rates have risen by 2–3% over the past 20 years, which is likely due to the increased detection of small primary tumors. In contrast to symptomatic tumors, incidentally diagnosed RCC primary and metastatic tumors are better candidates for definitive resection [1].

Surgical treatment remains the standard of care for localized, non-metastatic RCC, with the first report of radical nephrectomy in 1948 [2]. Initial surgical principles included kidney removal with its perirenal fat, regional lymph nodes, and ipsilateral adrenalectomy and open radical nephrectomy as the traditional *de facto* standard of care. Representative surgical series described long-term survival rates of 60%–90%, 20%–67%, 15%–80%, and 2%–20%, for stages I, II, III, and IV, respectively [3]. Surgical management has evolved as a result of emerging understanding of the biologic and clinical behavior of disease [4]. Contemporary studies suggest that the rate of adrenal metastases is low with no significant differences in cancer-specific survival rates with or without adrenalectomy. Similarly, the extent and need of routine lymphadenectomy continues to be debated. The Phase III EORTC 30881 [5], which compared radical nephrectomy with or without lymphadenectomy for stage T1-3N0M0 tumors, failed to detect a progression-free or survival benefit for lymphadenectomy at 5 years. In addition, indications for partial nephrectomy have expanded; modern long-term outcomes support its use as an effective alternative to radical nephrectomy with respect to cancer-specific survival. Extension into the inferior vena cava, which represents a significant challenge from a technical perspective, is advocated for patients with organ-confined tumors. Laparoscopic and more recently robotic resection approaches have emerged as alternatives to open nephrectomy in the management of properly selected patients with localized RCC without renal vein involvement or lymphadenectomy. Ablative techniques, with varying ranges of clinical experience including cryotherapy, radiofrequency ablation, microwave thermotherapy, and high-intensity focused ultrasound, are also being evaluated. The role of metastasectomy and/or cytoreductive nephrectomy in patients with metastatic disease remains controversial, although it is considered for palliation in patients with only partial regression of metastases or in those with prolonged disease regression following systemic therapy. One possible exception is the use of metastasectomy for solitary metastases, which can provide long-term remission rates of up to 30%, particularly in cases of metachronous pulmonary metastases.

Attempts at incorporating conventionally fractioned radiation into the routine management of RCC date back at least 50 years. Initial retrospective data described an apparent survival advantage with preoperative radiation *vs.* surgery alone; nevertheless, two subsequent prospective clinical trials failed to confirm such benefit. In the first, van der Werf-Messing *et al.* reported a series of 126 evaluable patients testing preoperative radiation to 30 Gy in 15 fractions followed by immediate nephrectomy and revealed no significant survival benefit but noted a possible benefit in patients with T3 lesions conferred by increased complete resection rates [6]. A second study by Juusela *et al.* utilized preoperative radiation at 2.2 Gy per fraction to 33 Gy with inferior overall survival (OS) for patients treated with preoperative radiotherapy (47% *vs.* 63% at 5 years) [7]. Similarly, postoperative radiotherapy failed to show a substantial benefit despite initial encouraging retrospective reports. A prospective randomized trial of postoperative radiotherapy conducted by the Copenhagen Renal Cancer Study Group compared nephrectomy alone to postoperative radiotherapy consisting of 50 Gy in 20 fractions, again without significant survival benefit (5-year survival 62% for nephrectomy alone *vs.* 38% nephrectomy and adjuvant radiotherapy), and noted significant complications in 44% of radiotherapy (RT) patients [8]. Emerging evidence suggests that the suboptimal efficacy of conventionally fractionated RT in the management of RCC stems from its unique biologic characteristics, of which salient findings are summarized in the following section.

## 2. Biology of Renal Cell Cancer—A Radiotherapeutic Perspective

Like breast, prostate, and colon cancers, RCC occurs in familial and sporadic forms [3]. Substantial clinical and laboratory investigation of RCC over the past two decades has led to expanded insight into the tumor biology and genetic basis. Notable gains in the understanding of the genetic basis and underlying biology of RCC branch from the investigation of the hereditary forms of the tumor [3]. RCC is associated with multiple syndromes, including von Hippel Lindau syndrome (VHL), tuberous sclerosis, hereditary papillary RCC, Britt-Hogg-Dubé (BHD) syndrome, and hereditary renal carcinoma. Recent advances in the biology of RCC demonstrate VHL-associated molecular features. For example, Gordan *et al.* described the effects of VHL tumor suppressor loss in hypoxia inducible factor alpha (HIF-α) stabilization as occurring in 70% of sporadic clear cell RCC [9]. His group analyzed VHL genotype and HIF-α expression in 160 primary tumors, which were segregated into three groups with distinct molecular characteristics. Tumors with intact VHL and those with VHL-deficiency but expressed HIF-1α and HIF-2α exhibited enhanced Akt/mTOR and ERK/MAP kinase signaling, whereas VHL-deficient RCCs that only expresses HIF-2α displayed elevated C-MYC activity. Therefore, the end effect of VHL on the HIF transcription factor profile may dictate tumor biology critical to therapeutic response or overall disease behavior.

Recent *in vitro* studies have investigated the relationship between RCC radioresistance and HIF-2α. Bhatt *et al.* examined cell lines expressing stable short-hairpin RNAs (shRNAs) encoding HIF-2α [10]. Cell lines were assayed for their response to increasing doses of ionizing radiation. Results demonstrated that RCC lines with decreased HIF-2α levels showed a significant increase in radiation sensitivity and an increase in G2 cell cycle arrest. Rapamycin, while effective in decreasing HIF1alpha protein levels, did not affect HIF2alpha levels in either of the RCC cell lines. The authors concluded that HIF-2α levels were inversely correlated to radiosensitivity, potentially accounting for clinical RCC radioresistance. In addition, the authors suggested that mTOR inhibitors might be ineffective radiosensitizers given their lack of effect on HIF-2α levels.

In addition to VHL and HIF, a number of genes have been implicated in the initiation, development, and progression of RCC. These include the FHIT and RASF1 tumor suppression genes, several forms of the TGF-β receptor, pentaerythritol tetranitrate, vascular endothelial growth factor (VEGF), and carbonic anhydrase.

The recent advances into our understanding of the molecular pathogenesis of RCC have translated directly into improved treatment options and oncologic outcomes, particularly for patients with metastatic disease, and exemplified by an ever-expanding array of targeted therapies [11]. Among these, VEGF receptor tyrosine kinase inhibitors and inhibitors of the mTOR signaling pathway have emerged as first-line options in the management of metastatic renal cell carcinoma (MRCC) based on improved progression-free and/or OS outcomes. Notably, as compared with interferon in patients with good or-intermediate risk clear renal cell carcinoma (CCRCC), the tyrokinase inhibitor sunitinib demonstrated an OS benefit (26.4 *vs.* 21.8 months, p = 0.051) even in the setting of crossover. Additionally, the tyrokinase inhibitors sorafenib and pazopanib have also received Food and Drug Administration approval. Current research efforts are directed towards understanding and overcoming the resistance mechanisms following initial response to therapy [12,13].

Beyond the VEGF pathway, inhibition of the mTOR signaling provides an alternate mechanism for targeted therapy in RCC. In the front-line setting, the mTOR inhibitor temsirolimus is utilized in patients with poor-risk disease given improved OS. A phase III, placebo-controlled study established a role for second-line treatment with the mTOR inhibitor everolimus by demonstrating a significant progression-free survival (PFS) benefit with a hazard ratio of 0.3, which prompted the approval of the agent following disease progression after VEGF inhibitor therapy [14].

Combination strategies, including “*vertical inhibition*,” which describes a combination of therapies that target factors working in a linear signaling pathway, and “*lateral inhibition*” which implies inhibiting targets from known overlapping pathways, both constitute foci of ongoing research studies with significant potential for integration of stereotactic radiotherapy (e.g., in the treatment oligometastases) as a local targeted therapeutic option.

Renal cell carcinoma has traditionally been considered intrinsically radioresistant although the specific molecular mechanisms responsible for such radioresistance have not been elucidated. However, radioresistance was verified through laboratory experiments by Deschavanne *et al.* indicating that RCC is amongst the most radioresistant *in vitro* cell types [15]. A possible explanation may lie in STAT1, a transcription factor downstream of the interferon signaling pathway. Recent data from Hui *et al.* demonstrated increased radiosensitivity amongst human CCRCC samples by the inhibition of STAT1 expression by fludarabine and siRNA [16]. The manipulation of this, and potentially additional pathways, may help identify agents allowing for synergistic activity in combination with RT, further expanding local management options.

Current radiobiologic modeling of dose response using conventional fraction sizes of 1.8–3 Gy is based on the concept of biologic effective dose (BED) [15,17]:

$$\text{BED}=nd\left(1+\frac{d}{\alpha /\beta }\right)$$

Two retrospective analyses have utilized the linear quadratic (LQ) formalism to investigate the biologic effective dose for appropriate palliative management of RCC. In the first, using a retrospective dataset of 107 patients and 150 irradiated sites, DiBiase *et al.* revealed an 86% palliative response rate after RT, including a 49% complete response rate [18]. Multivariate analysis identified performance status and a higher BED as significant response predictors. Nevertheless, the study utilized a presumed α/β value 10, which might be an overestimate based on more recent studies. A subsequent study by Wilson *et al.* evaluated 143 palliative treatments amongst 78 patients with MRCC and described a similar response rate of 73% [19]. The LQ formalism was used to calculate biologic effective doses using α/β ratios of 3 and 7. Response type and duration were not predicted by BED_3_ or BED_7_.

The efficacy of conventionally fractionated palliative radiotherapy for MRCC was best documented in a well-conducted, prospective phase II trial by Lee *et al.* which included 31 patients [20]. The authors prescribed 30 Gy in 10 fractions and documented pain, analgesic use, symptoms, and quality of life using validated questionnaire instruments prior to and at multiple times after RT. Among patients treated for pain, 83% experienced site-specific pain relief after RT, and 48% did not require increased analgesic medication. Unfortunately, global pain and quality of life scores were limited due to progressive systemic disease. Interestingly, the aforementioned response rate of 83% was achieved despite a BED_10_ of 39 Gy, which is lower than the threshold of 50 previously suggested by DiBiase *et al.* [18]. As a result, the authors supported the continued use of 30 Gy in 10 fractions as a palliative schedule for RCC, although their low patient numbers and limited follow-up evaluation may preclude definitive conclusions, particularly in the modern area of protracted survival of MRCC and sequential use of multiple targeted agents.

Despite the inherent controversies in optimizing RT management of MRCC relating to the aforementioned laboratory and clinical evidence supporting dose escalation, technical developments in the fields of stereotactic radiosurgery (SRS), which was utilized initially for brain tumors and most recently expanded to extracranial sites, have allowed RT treatment intensification with permissible toxicity rates [18]. The pertinent studies relating to cranial and extracranial radiosurgery for RCC will be described in the subsequent sections.

## 3. Radiotherapeutic Management of RCC Brain Metastases

Brain metastases constitute an important cause of morbidity and mortality in patients with RCC with reported incidence of brain metastases in 10% of patients based on an autopsy series. Over several decades, whole brain radiotherapy (WBRT) has constituted a standard treatment option in the management of brain metastases, in particular for the treatment of lung and breast cancer brain metastases, although it is potentially associated with neurocognitive dysfunction and with suboptimal control rates (especially for larger tumors) [21,22].

Traditional outcomes using WBRT for brain metastases in unselected patients are poor. An analysis from the Radiation Therapy Oncology Group (RTOG) database of prospective trials classified patients into three prognostic groups based on the recursive partitioning analysis (RPA) scheme with a median survival ranging from 2.3 to 7.1 months [23]. Outcomes after WBRT appear especially poor for patients with MRCC, a finding classically attributed to radioresistance. For example, a retrospective study by Wronski *et al.* revealed a median survival of 4.4 months from diagnosis following WBRT for RCC, with death from neurologic causes in 76% of patients [24]. These unsatisfactory results led to the proposal of more aggressive approaches, including surgical resection and radiosurgery, for RCC brain metastases.

Local treatment intensification in properly selected patients is sensible in light of published data. As an example, craniotomy for properly selected patients with solitary lesions is supported by the phase III trial by Patchell *et al.*, which demonstrated overall local PFS at the treated site as well as OS benefit (median time of 40 weeks in observation *vs.* 15 weeks in the WBRT-only arm) [25]. Similarly, technological advancements in the fields of radiotherapy and imaging dating back to the efforts of Leksell at the University of Uppsala in the 1950s have led to the development of intracranial stereotactic radiosurgery (SRS) techniques, which have been revised and optimized through several decades of development [26]. Originally employing narrow proton beams, practical considerations led investigators to the development of focused gamma- (*i.e.*, Gamma Knife) and X-ray- (*i.e.*, linear accelerator) based SRS technologies. Given the relative ease of frame-based immobilization, initial efforts focused on intracranial SRS. Conceptually, the technique describes an ablative use of therapeutic irradiation relying on convergence of multiple beams around a target volume, resulting in a heterogeneous dose distribution with sharp falloff gradients into surrounding normal tissue omitting prophylactic treatment of areas and subclinical risk of disease. The technique of intracranial SRS has now matured through several decades and is routinely performed in the outpatient basis either with invasive (frame-based), and non-invasive (frameless) methods. Results from a number of centers indicate excellent local control rates for small MRCC lesions treated with SRS as summarized in Table 1.

Despite the noted improvements in tumor control, a number of clinically relevant questions remain in the management of RCC brain metastases, which include the use of adjuvant WBRT, the selection of patients for stereotactic radiosurgery *vs.* SRS, and prognostic factors following stereotactic treatment in RCC patients.

With respect to the initial selection of radiosurgery *vs.* resection, for medically operable patients, significant factors include tumor size, number of metastases, and presence of symptomatic peritumoral edema. Surgical resection for brain metastases offers a number of attractive options, including confirmation of pathology, rapid reversal of neurologic symptoms, absence of neurologic symptoms, and durable local control for selected patients, but this comes with potential disadvantages including delays in systemic therapy and operative morbidity and mortality with incidence of up to 13% in a series of 400 craniotomies [32]. RCC-specific craniotomy outcomes have been reported in at least three retrospective series and have favored the inclusion of patients with superficial, easily resectable tumors in patients with higher Karknofsky scores at presentation. In a review of 50 patients, Wronski *et al.* described local recurrences in nine patients with overall recurrence of 49% [33]. The authors recommended consideration of craniotomy for effective palliation in selected patients. In consideration of modern systemic treatments for RCC, surgery is advocated for young (<65 years) patients with accessible, superficial solitary lesions, good performance status, and controlled extracranial disease or alternatively for those with symptomatic large lesions in need for rapid symptomatic relief [34].

The RTOG protocol 90-05 established SRS dose-volume prescription criteria for safety based on tumor size with tumor doses of 24, 18, and 15 Gy recommended in a single fraction for lesions up to 2 cm, between 2 and 3 cm, and greater than 3 cm in diameter, respectively [35]. Due to volumetric expansion of irradiated volume as a cubic function of lesion radius, single-fraction radiosurgery is limited to lesions ≤4 cm. In addition, in an analysis of 80 patients by Shuto *et al.*, patients with RCC brain metastases treated with Gamma Knife radiosurgery demonstrated increased edema index for RCC metastases *vs.* comparable patients with breast and lung primary tumors (Table 1) [30]. While an overall tumor control rate of 84% was achieved, peritumoral edema was only controlled in 64% of patients compared to 80% for other histologic types. Twelve patients who underwent craniotomy demonstrated local control rate of 67% with surgery alone with relative ease of resection and control of edema and without significant bleeding. A marginal dose of 20 Gy was suggested to achieve tumor control, but a dose of 25 Gy or more is needed to control peritumoral edema. A second analysis by Muacevic *et al.* of 85 patients with 376 brain metastases prescribed a mean tumor dose of 21.2 ± 3.2 Gy, and local/distant tumor recurrences were treated by additional SRS (Table 1) [31]. Patients had an overall median survival of 11.1 months, and local tumor control rate after SRS was 94%. While most (78%) of patients died from systemicx transient radiogenic complications, and three patients (3.5%) died because of intratumoral bleeding. Long-term reports conclusively document excellent intracranial local tumor control rates with SRS. Payne *et al.* described a retrospective series with 21 patients and 37 metastatic lesions treated with Gamma Knife (Table 1) [27]. In that early experience, 23 tumors were observed post-treatment, and none had progression. No radiation-induced changes in follow-up imaging were seen at an average of 21 months post-treatment. Sheehan *et al.* provided an update of the University of Pittsburgh series with median doses to the tumor margin of 16 Gy with a median OS of 15 months [36]. Radiographic local tumor control was seen in 96% of the patients. The quoted median survival time greatly exceeded that of the RTOG, RPA class I experience. Similarly Noel *et al.* also obtained excellent local control rates of 93% at 12 months using linac-based stereotactic radiosurgery with mean minimal dose of 14.7 Gy (Table 1) [28]. More recent studies have attempted to predict radiosurgical outcomes on the basis of early post-treatment imaging characteristics. Kim *et al.* defined good response as volumetric reduction of brain metastases to less than 75% of the baseline and noted a doubling of median survival times (18 months *vs.* 9 months, respectively), among good responders [37]. An updated analysis from the University of Pittsburgh revealed long-term local tumor control in 92% of patients treated with SRS with symptomatic adverse radiation effects in 7% (Table 1) [29]. Seventy percent of patients improved or remained neurologically stable. Multivariate analysis found that younger age, fewer brain metastases, absence of prior WBRT, and no prior systemic treatment (chemotherapy and/or immunotherapy) were associated with longer survival times. Salvage therapies utilized upon intracranial progression included repeat stereotactic radiosurgery, craniotomy, and WBRT. Six of 158 patients developed hemorrhage after SRS, and three required craniotomy. The topic of whether WBRT should be added to SRS for RCC patients remains controversial, particularly given the inconclusive data from randomized trials for patients with predominantly radiosensitive histologies including those by Patchell, Aoyama, and Kocher [25,38,39].

In aggregate, these studies suggested improved local and distant brain tumor control without demonstrable survival benefit for WBRT. Specifically for RCC, the retrospective series of 88 patients by Fokas *et al.* using a combination of SRS and/or WBRT for brain metastases also demonstrated improved intracranial control with WBRT without OS benefit [40]. In light of: (a) high local tumor control rates achievable with SRS for appropriate candidates; (b) ease of radiographic follow-up; and (c) in consideration of the potential for neurocognitive decline associated with WBRT, we recommend initial SRS followed by observation for most patients with limited (1 to 4) RCC brain metastases [41].

## 4. Extracranial Stereotactic Radiotherapy

While conventionally fractionated external beam radiation was investigated in the neoadjuvant, adjuvant and palliative roles, control rates (particularly for brain metastases and primary tumors) have been disappointing and have not produced survival improvement. Fortunately, cranial SRS has demonstrated excellent response rates for RCC metastases, and this has coincided with technical advances in imaging and immobilization techniques permitting the implementation of extracranial stereotaxy. The historical development of extracranial SRS/SBRT has been previously summarized [42]. Briefly, SBRT was developed in the mid-1990s at the Karolinska Institute in Sweden using a “stereotactic body frame” and rigid immobilization, and it was later adopted by Japanese and North American investigators [43,44]. In view of highly promising initial results for lung SBRT at the University of Indiana, the technique has been adopted by the RTOG, and the encouraging local control rates for patients with medically inoperable lung cancer were validated in a recent Phase II trial, which demonstrated primary tumor control rate of 98% at 3 years [45].

Similar to intracranial SRS, SBRT typically delivers high doses per fraction. Frameless systems that utilize volumetric (kilo-and-megavoltage, planar, and/or CT) image guidance as an integral component of therapy are now available. Abdominal and retroperitoneal targets are amenable for treatment, with breath-hold and gating techniques employed in case of large breathing-induced tumor displacement [46]. SBRT requires high dose distribution conformality with rapid falloff beyond the prescribed planning target volume (PTV). Optimized dose-volume conformality parameters have been derived as part of the prospective series of clinical trials from the RTOG, in particular for lung targets [47]. Conceptually, these require minimization of both the high and intermediate isodose volumes. In practice, this is typically accomplished via conformal or intensity-modulated techniques with a large number of beam projections inclusive of non-coplanar fields.

The clinical application of SBRT for RCC is somewhat limited compared to lung or liver sites; nevertheless, experience in clinical application is now emerging. A study by Hillman *et al.* reported on the combination of sunitinib with tumor irradiation and soy isoflavones. This combination showed dramatic inhibition of tumor growth, disruption of the vasculature, and apoptosis of kidney tumors [48]. Beitler *et al.* reported on nine patients with non-metastatic RCC (including two patients with bilateral disease) treated definitively using conformal techniques to 40 Gy in five fractions [49]. Extended survival was noted in four of the nine patients, all of whom had small (<3.4 cm), none-negative, organ-confined lesions. More extensive experience was reported from the Karolinska Institute [50-53]. The most recent trial described the use of SBRT for lung, kidney, and adrenal sites [52]. The authors employed abdominal compression for reproducibility. Using a range of PTV prescription doses, local control was achieved in 98% of the treated lesions, although follow-up was limited to less than 6 months in 19% of patients. Overall survival was 32 months. Teh *et al.* demonstrated similar control rates amongst 14 patients with 23 extracranial MRCC lesions and two patients with inoperable, biopsy-proven RCC [54]. Using doses ranging from 24 to 48 Gy in 3–6 fractions, a local tumor control rate of 87% was observed. Preliminary data using carbon ion therapy also suggests promising control rates, albeit with slow clinical response rates, among RCC patients treated with definitive intent. Specifically, Nomiya *et al.* described 5-year local control and cause-specific survival rates of 100% and 100% among a cohort of 10 patients [55].

Favorable outcomes for SBRT in the treatment of RCC spinal metastases have also been reported. Gertzen *et al.* described 48 patients with 60 RCC spine metastases at various levels [56]. Treatment delivery utilized the robotic Cyberknife system. Mean maximum dose was 20 Gy. The volume of spinal cord exceeding 8 Gy was minimized, with mean of 0.64 cubic centimeters. Results demonstrated pain improvement in 90% of patients with tumor control in six of seven patients treated for radiographic progression.

In consideration of the previously reviewed literature on targeted therapy for RCC, the prior reports demonstrate significant oncologic merit in the treatment of oligometastatic disease [57]. In support of this concept, high-dose treatment may stimulate proliferation of quiescent cells, increasing sensitivity to systemic agents. Alternatively, enhanced radiosensitivity may be achieved through inhibition of radioresistance, potentially achievable through mTOR inhibitors as demonstrated *in vitro* in sarcomas and tumor vasculature [58], potentially through suppression of DNA double-stranded break repair [59]. Despite the strong scientific rationale in support of combined therapy, clinical experience remains limited to selected case series. In a recent case report, Kirova *et al.* described partial remission of a large retroperitoneal RCC recurrence using intensity-modulated helical tomotherapy (45 Gy in 25 fractions) and concurrent everolimus at 10 mg daily dosing [60]. Questions remain regarding the optimal dose and fractionation schemes for SBRT of RCC (in particular, with respect to minimum doses required for control of oligometastases). A recent analysis by Stinauer *et al.* evaluated SBRT outcomes in patients with metastatic melanoma (n = 17 patients, 28 lesions) or RCC (n = 13 patients, 25 lesions) [61]. The SBRT dose regimen was converted to the single fraction equivalent dose (SFED) for dose-response analysis. Various SBRT regimens were employed, ranging from 40–60 Gy in 3–5 fractions. At a median follow-up of 28 months for living patients, actuarial local control was 88% at 18 months. Results suggested optimal outcomes for larger fraction sizes and SFED regimens exceeding 45 Gy, with comparable control rates with respect to “classically radiosensitive” histologies. Their findings are in need of further validation.

## 5. Conclusions

As demonstrated in this review, the role of radiation therapy in RCC is bounded by its intrinsic radiobiologic properties conferring radioresistance to conventionally fractionated radiotherapy in the range of 1.8–3 Gy per fraction with a possible exception of symptomatic pain relief for selected bony metastases. Nevertheless, the advent of intra- and extra-cranial stereotactic techniques have ushered a promising era with a broadened scope for radiation therapy in primary unresectable renal tumors as well as intra- and extra-cranial metastases. These developments have fortunately coincided with advances in systemic therapy, which have resulted in improved OS rates for metastatic disease. Research efforts are underway to explore the optimal sequencing of combined SBRT and systemic therapy for oligometastases.

## Figures and Tables

**Table 1.** Selected SRS series in RCC brain metastases.

**Author**	**No. of Lesions Treated**	**SRS Type**	**Median Marginal Dose (Gy)**	**Tumor Control Rate (%)**	**Distant Failure Rate (%)**	**Median Survival (m)**	**Death From Brain Disease (%)**
Payne *et al.* [27]	37	GK	20	100	50	8	0
Noel *et al.* [28]	65	LINAC	17.3	97	N/A	11	29
Muacevic *et al.* [29]	376	GK	21.2	94	33	11.1	10
Shuto *et al.* [30]	444	GK	22	84	39	12	17.2
Kano *et al.* [31]	531	GK	18	92	36	8.2	N/A

## References

[1] Thompson I.M., Peek M. (1988). Improvement in survival of patients with renal cell carcinoma-the role of the serendipitously detected tumor. J. Urol..

[2] Mortensen H. (1948). Transthoracic nephrectomy. J. Urol..

[3] Gunderson L., Tepper J. (2007). Clinical Radiation Oncology.

[4] Lam J.S., Breda A., Belldegrun A.S., Figlin R.A. (2006). Evolving principles of surgical management and prognostic factors for outcome in renal cell carcinoma. J. Clin. Oncol..

[5] Blom J.H.M., van Poppel H., Maréchal J.M., Jacqmin D., Schröder F.H., de Prijck L., Sylvester R. (2009). The EORTC Genitourinary Tract Cancer Group. Radical nephrectomy with and without lymph-node dissection: Final results of European organization for research and treatment of cancer (EORTC) randomized phase 3 trial 30881. Eur. Urol..

[6] van der Werf-Messing B. (1973). Proceedings: Carcinoma of the kidney. Cancer.

[7] Juusela H., Malmio K., Alfthan O., Oravisto K.J. (1977). Preoperative irradiation in the treatment of renal adenocarcinoma. Scand. J. Urol. Nephrol..

[8] Kjaer M., Iversen P., Hvidt V., Bruun E., Skaarup P., Hansen J.B., Frederiksen P.L. (1987). A randomized trial of postoperative radiotherapy *versus* observation in stage II and III renal adenocarcinoma. A study by the Copenhagen Renal Cancer Study Group. Scand. J. Urol. Nephrol..

[9] Gordan J.D., Lal P., Dondeti V.R., Letrero R., Parekh K.N., Oquendo C.E., Greenberg R.A., Flaherty K.T., Rathmell W.K., Keith B. (2008). Hif-alpha effects on c-myc distinguish two subtypes of sporadic VHL-deficient clear cell renal carcinoma. Cancer Cell.

[10] Bhatt R.S., Landis D.M., Zimmer M., Torregrossa J., Chen S., Sukhatme V.P., Iliopoulos O., Balk S., Bubley G.J. (2008). Hypoxia-inducible factor-2α: Effect on radiationsensitivity and differential regulation by an mTOR inhibitor. BJU Int..

[11] Rathmell W.K., Godley P.A. (2010). Recent updates in renal cell carcinoma. Curr. Opin. Oncol..

[12] Rini B.I., Atkins M.B. (2009). Resistance to targeted therapy in renal-cell carcinoma. Lancet Oncol..

[13] Motzer R.J., Hutson T.E., Tomczak P., Michaelson M.D., Bukowski R.M., Oudard S., Negrier S., Szczylik C., Pili R., Bjarnason G.A. (2009). Overall survival and updated results for sunitinib compared with interferon alfa in patients with metastatic renal cell carcinoma. J. Clin. Oncol..

[14] Motzer R.J., Escudier B., Oudard S., Hutson T.E., Porta C., Bracarda S., Grünwald V., Thompson J.A., Figlin R.A., Hollaender N. (2008). Efficacy of everolimus in advanced renal cell carcinoma: A double-blind, randomised, placebo-controlled phase III trial. Lancet.

[15] Deschavanne P.J., Fertil B. (1996). A review of human cell radiosensitivity *in vitro*. Int. J. Radiat. Oncol. Biol. Phys..

[16] Hui Z., Tretiakova M., Zhang Z., Li Y., Wang X., Zhu J.X., Gao Y., Mai W., Furge K., Qian C.N. (2009). Radiosensitization by inhibiting STAT1 in renal cell carcinoma. Int. J. Radiat. Oncol. Biol. Phys..

[17] Fowler J.F. (1989). The linear-quadratic formula and progress in fractionated radiotherapy. Br. J. Radiol..

[18] DiBiase S.J., Valicenti R.K., Schultz D., Xie Y., Gomella L.G., Corn B.W. (1997). Palliative irradiation for focally symptomatic metastatic renal cell carcinoma: Support for dose escalation based on a biological model. J. Urol..

[19] Wilson D., Hiller L., Gray L., Grainger M., Stirling A., James N. (2003). The effect of biological effective dose on time to symptom progression in metastatic renal cell carcinoma. Clin. Oncol. (R. Coll. Radiol.).

[20] Lee J., Hodgson D., Chow E., Bezjak A., Catton P., Tsuji D., O'Brien M., Danjoux C., Hayter C., Warde P. (2005). A phase II trial of palliative radiotherapy for metastatic renal cell carcinoma. Cancer.

[21] Saitoh H., Shimbo T., Tasaka T., Iida T., Hara K. (1982). Brain metastasis of renal adenocarcinoma. Tokai J. Exp. Clin. Med..

[22] Khuntia D., Brown P., Li J., Mehta M.P. (2006). Whole-brain radiotherapy in the management of brain metastasis. J. Clin. Oncol..

[23] Gaspar L., Scott C., Rotman M., Asbell S., Phillips T., Wasserman T., McKenna W.G., Byhardt R. (1997). Recursive partitioning analysis (RPA) of prognostic factors in three radiation therapy oncology group (RTOG) brain metastases trials. Int. J. Radiat. Oncol. Biol. Phys..

[24] Wrónski M., Maor M.H., Davis B.J., Sawaya R., Levin V.A. (1997). External radiation of brain metastases from renal carcinoma: A retrospective study of 119 patients from the MD Anderson Cancer Center. Int. J. Radiat. Oncol. Biol. Phys..

[25] Patchell R.A., Tibbs P.A., Regine W.F., Dempsey R.J., Mohiuddin M., Kryscio R.J., Markesbery W.R., Foon K.A., Young B. (1998). Postoperative radiotherapy in the treatment of single metastases to the brain: A randomized trial. JAMA.

[26] Leksell L. (1951). The stereotaxic method and radiosurgery of the brain. Acta Chir. Scand..

[27] Payne B.R., Prasad D., Szeifert G., Steiner M., Steiner L. (2000). Gamma surgery for intracranial metastases from renal cell carcinoma. J. Neurosurg..

[28] Noel G., Valery C.A., Boisserie G., Cornu P., Hasboun D., Simon J.M., Tep B., Ledu D., Delattre J.Y., Marsault C. (2004). Linac radiosurgery for brain metastasis of renal cell carcinoma. Urol. Oncol..

[29] Muacevic A., Kreth F.W., Mack A., Tonn J.C., Wowra B. (2004). Stereotactic radiosurgery without radiation therapy providing high local tumor control of multiple brain metastases from renal cell carcinoma. Minim. Invasive Neurosurg..

[30] Shuto T., Matsunaga S., Suenaga J., Inomori S., Fujino H. (2010). Treatment strategy for metastatic brain tumors from renal cell carcinoma: Selection of gamma knife surgery or craniotomy for control of growth and peritumoral edema. J. Neurooncol..

[31] Kano H., Iyer A., Kondziolka D., Niranjan A., Flickinger J.C., Lunsford L.D. (2011). Outcome predictors of gamma knife radiosurgery for renal cell carcinoma metastases. Neurosurgery.

[32] Sawaya R., Hammoud M., Schoppa D., Hess K.R., Wu S.Z., Shi W.M., Wildrick D.M. (1998). Neurosurgical outcomes in a modern series of 400 craniotomies for treatment of parenchymal tumors. Neurosurgery.

[33] Wroński M., Arbit E., Russo P., Galicich J.H. (1996). Surgical resection of brain metastases from renal cell carcinoma in 50 patients. Urology.

[34] Remon J., Lianes P., Martínez S. (2011). Brain metastases from renal cell carcinoma. Should we change the current standard?. Cancer Treat. Rev..

[35] Shaw E., Scott C., Souhami L., Dinapoli R., Kline R., Loeffler J., Farnan N. (2000). Single dose radiosurgical treatment of recurrent previously irradiated primary brain tumors and brain metastases: Final report of rtog protocol 90-05. Int. J. Radiat. Oncol. Biol. Phys..

[36] Sheehan J.P., Sun M.H., Kondziolka D., Flickinger J., Lunsford L.D. (2003). Radiosurgery in patients with renal cell carcinoma metastasis to the brain: Long-term outcomes and prognostic factors influencing survival and local tumor control. J. Neurosurg..

[37] Kim W.H., Kim D.G., Han J.H., Paek S.H., Chung H.T., Park C.K., Kim C.Y., Kim Y.H., Kim J.W., Jung H.W. (2011). Early significant tumor volume reduction after radiosurgery in brain metastases from renal cell carcinoma results in long-term survival. Int. J. Radiat. Oncol. Biol. Phys..

[38] Aoyama H., Shirato H., Tago M., Nakagawa K., Toyoda T., Hatano K., Kenjyo M., Oya N., Hirota S., Shioura H. (2006). Stereotactic radiosurgery plus whole-brain radiation therapy *vs.* stereotactic radiosurgery alone for treatment of brain metastases: A randomized controlled trial. JAMA.

[39] Kocher M., Soffietti R., Abacioglu U., Villà S., Fauchon F., Baumert B.G., Fariselli L., Tzuk-Shina T., Kortmann R.D., Carrie C. (2011). Adjuvant whole-brain radiotherapy *versus* observation after radiosurgery or surgical resection of one to three cerebral metastases: Results of the EORTC 22952-26001 study. J. Clin. Oncol..

[40] Fokas E., Henzel M., Hamm K., Surber G., Kleinert G., Engenhart-Cabillic R. (2010). Radiotherapy for brain metastases from renal cell cancer: Should whole-brain radiotherapy be added to stereotactic radiosurgery: Analysis of 88 patients. Strahlenther. Onkol..

[41] Chang E.L., Wefel J.S., Hess K.R., Allen P.K., Lang F.F., Kornguth D.G., Arbuckle R.B., Swint J.M., Shiu A.S., Maor M.H. (2009). Neurocognition in patients with brain metastases treated with radiosurgery or radiosurgery plus whole-brain irradiation: A randomised controlled trial. Lancet Oncol..

[42] Lo S.S., Fakiris A.J., Chang E.L., Mayr N.A., Wang J.Z., Papiez L., Teh B.S., McGarry R.C., Cardenes H.R., Timmerman R.D. (2010). Stereotactic body radiation therapy: A novel treatment modality. Nat. Rev. Clin. Oncol..

[43] Uematsu M., Shioda A., Tahara K., Fukui T., Yamamoto F., Tsumatori G., Ozeki Y., Aoki T., Watanabe M., Kusano S. (1998). Focal, high dose, and fractionated modified stereotactic radiation therapy for lung carcinoma patients: A preliminary experience. Cancer.

[44] Timmerman R., Papiez L., McGarry R., Likes L., DesRosiers C., Frost S., Williams M. (2003). Extracranial stereotactic radioablation: Results of a phase I study in medically inoperable stage I non-small cell lung cancer. Chest.

[45] Timmerman R., Paulus R., Galvin J., Michalski J., Straube W., Bradley J., Fakiris A., Bezjak A., Videtic G., Johnstone D. (2010). Stereotactic body radiation therapy for inoperable early stage lung cancer. JAMA.

[46] Teh B.S., Ishiyama H., Mathews T., Xu B., Butler E.B., Mayr N.A., Lo S.S., Lu J.J., Blanco A.I., Paulino A.C. (2010). Stereotactic body radiation therapy (SBRT) for genitourinary malignancies. Discov. Med..

[47] Timmerman R., Galvin J., Michalski J., Straube W., Ibbott G., Martin E., Abdulrahman R., Swann S., Fowler J., Choy H. (2006). Accreditation and quality assurance for Radiation Therapy Oncology Group: Multicenter clinical trials using stereotactic body radiation therapy in lung cancer. Acta Oncol..

[48] Hillman G.G., Singh-Gupta V., Al-Bashir A.K., Yunker C.K., Joiner M.C., Sarkar F.H., Abrams J., Haacke E.M. (2011). Monitoring sunitinib-induced vascular effects to optimize radiotherapy combined with soy isoflavones in murine xenograft tumor. Transl. Oncol..

[49] Beitler J.J., Makara D., Silverman P., Lederman G. (2004). Definitive, high-dose-per-fraction, conformal, stereotactic external radiation for renal cell carcinoma. Am. J. Clin. Oncol..

[50] Wersäll P.J., Blomgren H., Lax I., Kälkner K.-M., Linder C., Lundell G., Nilsson B., Nilsson S., Näslund I., Pisa P. (2005). Extracranial stereotactic radiotherapy for primary and metastatic renal cell carcinoma. Radiother. Oncol..

[51] Wersäll P.J., Blomgren H., Pisa P., Lax I., Kälkner K.-M., Svedman C. (2006). Regression of non-irradiated metastases after extracranial stereotactic radiotherapy in metastatic renal cell carcinoma. Acta Oncol..

[52] Svedman C., Karlsson K., Rutkowska E., Sandström P., Blomgren H., Lax I., Wersäll P. (2008). Stereotactic body radiotherapy of primary and metastatic renal lesions for patients with only one functioning kidney. Acta Oncol..

[53] Svedman C., Sandström P., Pisa P., Blomgren H., Lax I., Kälkner K.-M., Nilsson S., Wersäll P. (2006). A prospective phase II trial of using extracranial stereotactic radiotherapy in primary and metastatic renal cell carcinoma. Acta Oncol..

[54] Teh B., Bloch C., Galli-Guevara M., Doh L., Richardson S., Chiang S., Yeh P., Gonzalez M., Lunn W., Marco R. (2007). The treatment of primary and metastatic renal cell carcinoma (RCC) with image-guided stereotactic body radiation therapy (SBRT). Biomed. Imaging Interv. J..

[55] Nomiya T., Tsuji H., Hirasawa N., Kato H., Kamada T., Mizoe J., Kishi H., Kamura K., Wada H., Nemoto K. (2008). Carbon ion radiation therapy for primary renal cell carcinoma: Initial clinical experience. Int. J. Radiat. Oncol. Biol. Phys..

[56] Gerszten P.C., Burton S.A., Ozhasoglu C., Vogel W.J., Welch W.C., Baar J., Friedland D.M. (2005). Stereotactic radiosurgery for spinal metastases from renal cell carcinoma. J. Neurosurg..

[57] Lo S.S., Fakiris A.J., Teh B.S., Cardenes H.R., Henderson M.A., Forquer J.A., Papiez L., McGarry R.C., Wang J.Z., Li K. (2009). Stereotactic body radiation therapy for oligometastases. Expert Rev. Anticancer Ther..

[58] Murphy J.D., Spalding A.C., Somnay Y.R., Markwart S., Ray M.E., Hamstra D.A. (2009). Inhibition of mTOR radiosensitizes soft tissue sarcoma and tumor vasculature. Clin. Cancer Res..

[59] Chen H., Ma Z., Vanderwaal R.P., Feng Z., Gonzalez-Suarez I., Wang S., Zhang J., Roti J.L.R., Gonzalo S., Zhang J. (2011). The mTOR Inhibitor rapamycin suppresses DNA double-strand break repair. Radiat. Res..

[60] Kirova Y.M., Servois V., Chargari C., Amessis M., Zerbib M., Beuzeboc P. (2010). Further developments for improving response and tolerance to irradiation for advanced renal cancer: Concurrent (mTOR) inhibitor RAD001 and helical tomotherapy. Invest. New Drugs.

[61] Stinauer M.A., Kavanagh B.D., Schefter T.E., Gonzalez R., Flaig T., Lewis K., Robinson W., Chidel M., Glode M., Raben D. (2011). Stereotactic body radiation therapy for melanoma and renal cell carcinoma: Impact of single fraction equivalent dose on local control. Radiat. Oncol..

